# The regulatory mechanisms of proline and hydroxyproline metabolism: Recent advances in perspective

**DOI:** 10.3389/fonc.2022.1118675

**Published:** 2023-01-26

**Authors:** James M. Phang

**Affiliations:** Mouse Cancer Genetics Program, Center for Cancer Research, National Cancer Institute at Frederick (NIH), Frederick, MD, United States

**Keywords:** shared enzymes, competition, hydroxylation, CSC, demethylation

## Abstract

For diverse human tumors, growth and metastasis are dependent on proline synthesis, but the mechanisms underlying this association are not clear. Proline incorporated into collagen is primarily synthesized from glutamine. Thus, rates of collagen synthesis are modulated by the enzymes of proline synthesis. On the other hand, the hydroxylation of collagen proline requires αKG, ascorbate and ferrous iron, substrates necessary for the epigenetic demethylation of DNA and histones. The metabolic relationship of proline and hydroxyproline degradation are initiated by distinct dehydrogenases but the respective oxidized products, P5C and OH-P5C are substrates for P5C Reductase and P5C Dehydrogenase allowing for mutual competition. This provides a model by which proline synthesis in cancer plays a role in reprogramming gene expression. The metabolism of proline and hydroxyproline are also linked to the HIF response to hypoxia. Hypoxia increased the expression of ALDH18A1, which is the limiting step in proline and collagen synthesis. Hydroxyproline increases levels of HIF-1α presumably by inhibiting its degradation. These new findings allow the suggestion that there is a regulatory axis from glutamine to proline and collagen synthesis, and the release of free hydroxyproline can feed back on the HIF pathway.

## Introduction

After the initial emphasis on glucose and glutamine in cancer metabolism, interest shifted to other substrates, e.g., amino acids and lipids. The original hypothesis for the “Warburg Effect,” was to conserve carbon moieties for cell mass rather than being consumed by oxidative phosphorylation for energy (1). Similarly, the reprogramming of amino acid metabolism was thought to channel amino acids into proteinogenesis or to interconvert amino acids to safeguard the building blocks for proteins (2). A few special functions of amino acid metabolism were identified to furnish functions necessary for cellular proliferation or to mediate special metabolic pathways. These include the serine/glycine pathway for methyl transfers, glutamine to furnish amino groups for purine and pyrimidine nucleotides and branched-chain amino acids for lipogenesis (3). Especially interesting are the non-essential amino acids (NEAAs) where the biosynthetic mechanisms making them nonessential provide critical contributions to metabolism. An important metabolic system often omitted from reviews of amino acid metabolism in cancer is that for the amino acid proline (4, 5). This omission may be due to the complexities of proline metabolism, its interaction with the metabolism of hydroxyproline, and the critical function of proline in the synthesis of collagen thereby forming the infrastructure for the microenvironment. Additionally, hydroxylation of protein prolines, a metabolic reaction central to the formation of collagen, also is the mechanism responding to hypoxia (6) and also may play an essential step in metabolic epigenetics.

The features of proline synthesis and degradation were recognized since no other amino (imino) acid had a common metabolite which served as direct precursor as well as immediate degradative product (4). Due to this relationship of metabolites, a “proline cycle” was recognized. Additionally, the metabolite, pyrroline-5-carboxlate (P5C) could transfer oxidizing potential into cells to activate certain pathways, i.e., the pentose phosphate pathway (PPP) and markedly increase the formation of ribose, PRPP and nucleotides (7). The induction of proline dehydrogenase (PRODH) by P53, and the upregulation of the proline synthetic enzymes by MYC and by AKT and PI3K suggested that the proline metabolic system plays an important role in oncogenic proliferation and metabolic reprogramming (5). In clinical screening, increased PYCR1 is associated with many human cancers, (8) and knockdown of PYCR 1 in tumors transplanted into mice markedly decreased tumor growth. Additionally, the inhibition of the ALDH18A1- MYCN positive feed back loop attenuates MYCN-amplified neuroblastoma growth (9). Importantly, the availability of preformed proline does not mitigate the inability to synthesize proline suggesting either compartmentation of endogenously synthesized proline or a process channeling synthesized proline for the synthesis of collagen. This apparent routing of synthesized proline to collagen and the special metabolic role of collagen turnover has been recently reviewed (8). Furthermore, the mechanoregulatory properties of collagen and the role of Kindlin-2 and Pinch-1, two sensors of collagen stiffness caused upregulation of PYCR1 strongly suggesting that proline synthesis is linked to a functional role of collagen (10). These regulatory functions of proline metabolism and especially the relationship of collagen turnover to proline metabolism has been recently reviewed (8).

During the last 2 years, a number of new directions came from interesting findings from a number of laboratories. Although it would take a voluminous review to cover them all, I have taken the liberty of emphasizing a number of new and somewhat unexpected discoveries. These include: 1) The transitional pluripotency mediated by proline metabolism in embryonic stem cells (ESC) (11), and 2) also in cancer stemlike cells (CSC) (12); 3) The interaction between proline metabolism and hydroxyproline metabolism *(* 13) 4) the effect of hydroxyproline with HIF-1α (13); 5) the hydroxylation of collagen and its modulation of demethylation in TET and Jmj (8); 6) In CAR-T Lymphocytes, screened by gain-of-function CRISPR screens, the discovery that PRODH2 (hydroxyproline dehydrogenase) was the only gene which significantly activated the tumorlytic effect of CAR-T cells (14). We will discuss these subjects in order and attempt to explain the mechanisms and their implications using state-of-the-art insights into proline metabolism and cancer (11).

## The interaction between the metabolism of proline and hydroxyproline

**Figure 1:**
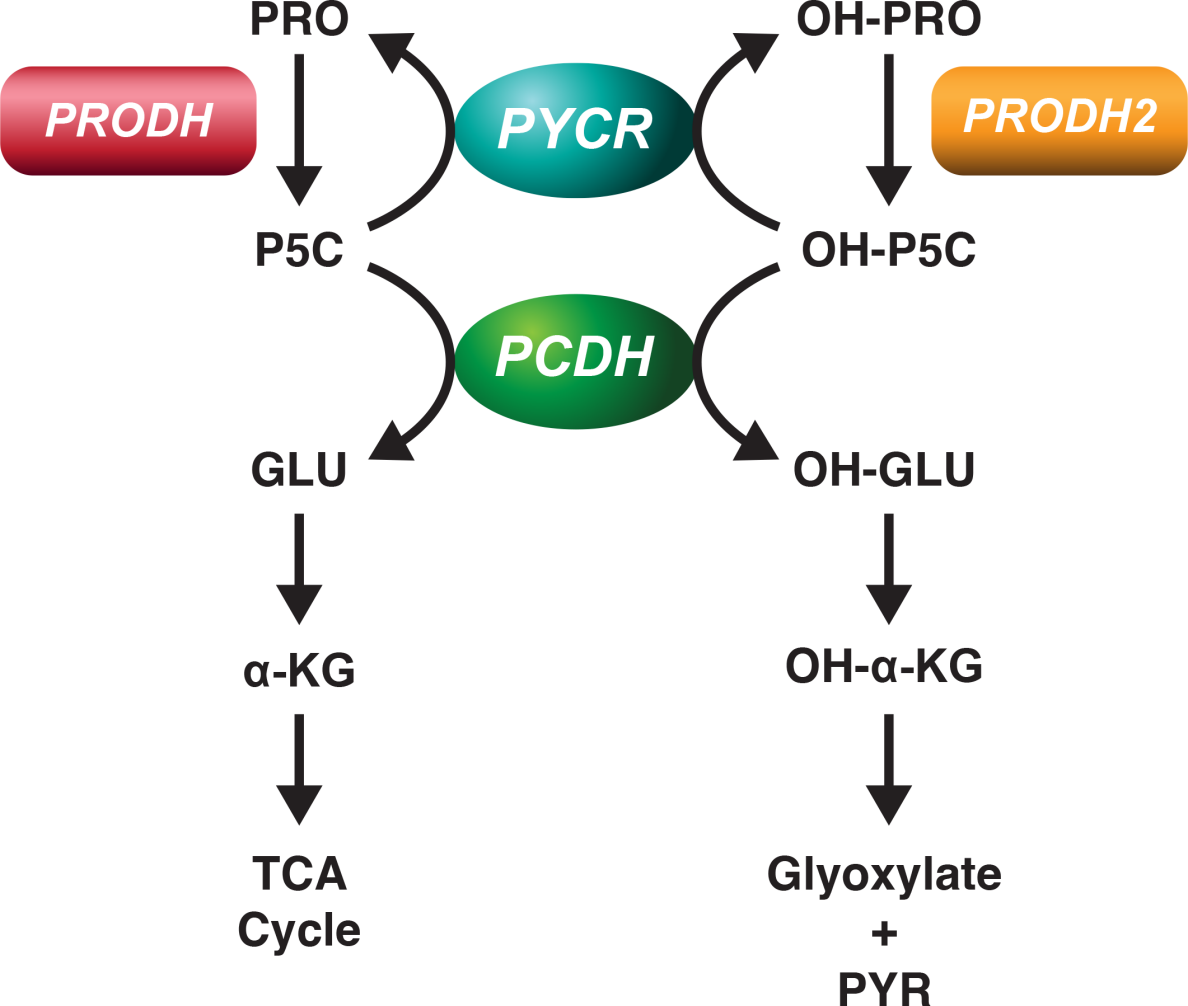
Metabolic Pathways for proline and hydroxyproline. Proline and hydroxyproline are metabolized by distinct dehydrogenases, the products are pyrroline-5-carboxylate and OH-pyrroline-5-carboxylate, respectively. Proline dehydrogenase (PRODH) and Hydroxyproline dehydrogenase (PRODH2) are distinct gene products but both are bound to mitochondrial inner membranes and donate electrons to FAD at site II. PCDH is in mitochondrial matrix and converts P5C and OH-P5C to glutamate and OH-glutamate, respectively. Both P5C and OH-P5C can be recycled to proline and hydroxyproline, respectively by generic PYCR. Since 3 isozymes of PYCR are known, preferential affinity of the isozymes for P5C and OH-P5C has not been defined. Competition between P5C and OH-P5C for PYCR1-3 and for PCDH has not been examined.

An interesting finding appeared from work in hepatocellular carcinoma. The workers showed that there was markedly accelerated consumption of proline with accumulation of hydroxyproline (13). They found that hypoxia Increased the expression of ALDH18Al, which codes for pyrroline-5-carboxylate synthase and increases proline formation. Importantly, accompanying hypoxia, an increase in hydroxyproline increased the level of HIIF-1a. Since HIF-1a levels are (13) collagen, it is not surprising that there may be interaction between hydroxyproline and the hydroxylation of HIF-1α (13). It is noteworthy that several authors back in the 1980s described the phenomenon of early intracellular degradation of collagen before it is secreted to form fibrils (15). Others have shown the differential growth sensitivity to 4-*cis*-Hydroxy-L-proline of transformed rodent cell lines (16). One interpretation of these previous findings is the suggestion that the production of free hydroxyproline is important for cellular regulation and the only way this can occur is through hydroxylation of proline in collagen. Thus, the release of free hydroxyproline may benefit the cell with the regulatory function of hydroxyproline (**Figure 1**). Although the metabolism of proline in cancer has received increasing attention, the metabolism of hydroxyproline has been reviewed (17), but the metabolic interaction between these two imino acids has received little attention.

From previous work we showed that there the metabolism of hydroxyproline is distinct from and yet shares certain pathways with proline (18). Clearly the first steps in degradation of proline and hydroxyproline, respectively, are catalyzed by proline dehydrogenase (PRODH) and hydroxyproline dehydrogenase (PRODH2). Mary Efron showed no detectible difference in PRODH activity or proline metabolism in patients with hydroxyprolinemia and no deficiency of PRODH (19). In assays for both proline dehydrogenase and hydroxyproline dehydrogenase, we could find little overlap in activities, 100 fold concentrations of one did not affect the metabolism of its congener (18).

On the other hand, at the level of both P5C reductase and P5C dehydrogenase, the enzymes which convert P5C to proline and glutamate, respectively, can use OH-P5C, the product of hydroxyproline dehydrogenase as substrate to produce hydroxyproline and hydroxyglutamate, respectively (**Figure 1**). This property of P5C reductase was first described by Adams and Goldstone (20) with partially purified protein, and the P5C dehydrogenase was discovered in patients with Type 2 hyperprolinemia who accumulated both P5C and OH-P5C (21) and excreted both compounds in urine. With this recognition, one would expect that they may mutually inhibit each other’s activity, but this requires direct measurements using P5C and OH-P5C. Importantly, in the case of P5C reductase, one may have to revise our understanding of the 3 isozymes which may have different affinities and be inhibited differentially by OH-P5C (22). In fact, it may be that a particular isozyme may be specific for OH-P5C rather than P5C, but this would require additional studies using P5C and/or OH-P5C as substrates.

## Proline metabolism mediates transitional pluripotency

During the last year, an important review was published by G. Minchiotti in Naples. She reviewed the original observation on mouse embryonic stem cells which was then extended by workers in her own laboratory (11, 23) who showed that treatment with proline preserved the pluripotent state. The mechanism underlying this effect may be due to the early primed state of pluripotency. Whether this effect is parallel to the reactivation of cancer stem-like cells has to be shown. A paper recently showed that knock down of PYCR1 and 2 effectively blocked the formation of mammospheres (12) in contradistinction to inhibitors of glutaminase which had little effect on mammosphere formation. This finding clearly defines that although glutamine serves as the main delivery source of degradation products of various amino acids and proteins, it is the synthesis of proline which is critical for forming mammospheres, an accepted criteria of renewal of cancer stem-like cells (24).

## Proline metabolism and the formation of collagen

It is well-recognized that type I collagen is the most abundant protein in the human body. If hydroxyproline is considered to be posttranslationally modified, one must recognize that proline incorporated into collagen makes up about 25% of amino acids incorporated. Unexpectedly, the proline incorporated into collagen by fibroblasts and CAF’s are not from the total intracellular PRO pool, but preferentially from synthesized proline (25–27). The hydroxylation of collagen proline by P4H forming hydroxyproline is the most abundant flux in the body. During the 1980s, several labs documented the early intracellular degradation of collagen before it was secreted to form fibrils (15), and the differential sensitivity to 4-*cis*-hydroxyproline (16) suggested that free hydroxyproline was an important regulatory metabolite which could only be formed by posttranslational modification and degradation.

## Function of collagen as the major constituent of ECM and the microenvironment

Collagen is thought to play an important role between cancer cells and their microenvironment (28). For human breast cancer, ovarian and pancreatic cancers, dense collagen is thought to wall off the cancer from immune surveillance. Additionally, collagen acts as a mechanoregulator. The molecular mechanism linking ECM stiffening to proline and collagen synthesis involve Pinch-1 and Kindlin-2 (10) which translocate and activates PYCR-1. Thus, the molecular players linking mechanoregulation to the proline/collagen regulatory axis are beginning to be understood.

## Collagen synthesis with hydroxylation of proline modulates activity of epigenetic dioxygenases

**Figure 2:**
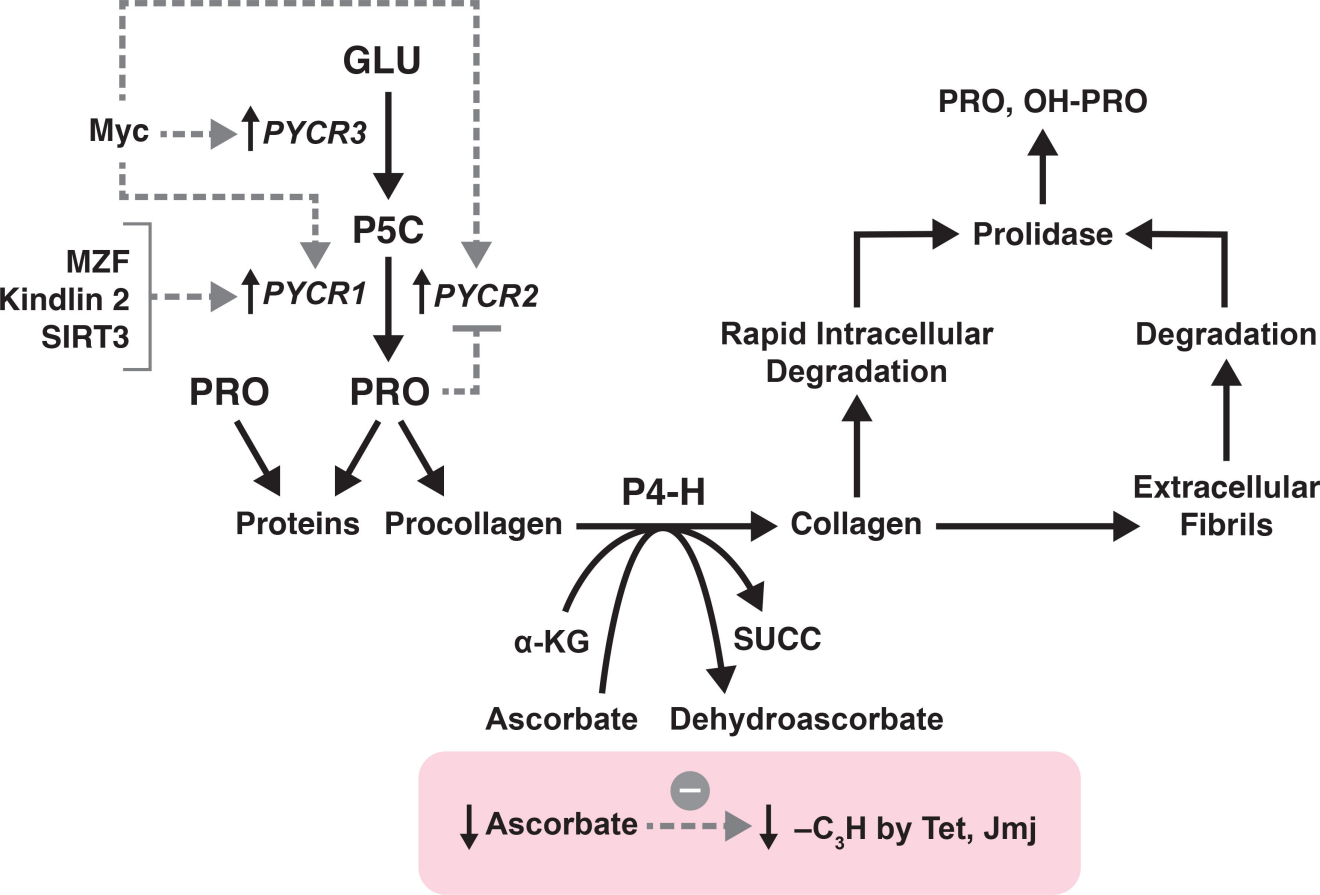
The metabolic link between proline synthesis, collagen formation, formation of free PRO and OH-PRO and epigenetics. Collagen formation by Cancer-associated fibroblasts is linked to biosynthesized proline. The hydroxylation of procollagen, by P4-H exhausts a-KG and ascorbate which decreases demethylation by Tet and Jmj. Free hydroxyproline is formed by early intracellular collagen degradation as well as by extracellular degradation of collagen fibrils. Prolidase is necessary for the hydrolysis of imidodipeptides, i.e. proline and hydroxyproline as C-terminal dipeptides.

The putative role of collagen metabolism in influencing metabolic epigenetics has been reviewed (8, 29). Because proline biosynthesis may be rate limiting for the incorporation of proline into collagen, the rate by which hydroxylation is formed can also be influenced. Since the hydroxylation mechanism involves a dioxygenase reaction involving oxygen, alpha KG, ascorbate and ferrous iron, competition for these substrates may have metabolic consequences (**Figure 2**). This linkage has been suggested as the metabolic influence underlying epigenetic regulation. The coupling of proline synthesis to collagen formation and modulation of metabolic epigenetic has been reviewed (8). The concept is that the hydroxylation mechanism of collagens, its use of α-KG, ascorbate, Fe++, is similar in substrate demand as the demethylases of DNA and histones, Tet and Jmj, respectively. Thus, synthesized proline can influence the rate of collagen synthesis and provide the metabolic linkage to regulate epigenetic mechanisms.

## PRODH2 has been found on genomic screen for activation of CAR-T cells

With the objective of increasing the anti-tumor activity of CAR-T cells, workers performed a gain-of-function genomic screen using CRISPR. Surprisingly, the gene uniquely augmenting CAR-T cells was PRODH2, i.e. hydroxyproline dehydrogenase (14). Although PRODH2 is important in the degradation of collagen, there has not been any suggestion that it could play a regulatory role specifically for immune mechanisms. Whether PRODH2 is normally expressed in lymphocytes with or without immunologic stimulation remains unknown. Although PRODH and PRODH2 produce pyrroline-5-carboxylate (P5C) and hydroxy-pyrroline-5-carboxylate (OH-P5C), respectively, they are encoded by distinct genes and there is little overlap or cross-inhibition of activity. The final products of the two degradative pathways are alpha-KG for proline and glyoxylate and pyruvate for hydroxyproline.

**Figure 3:**
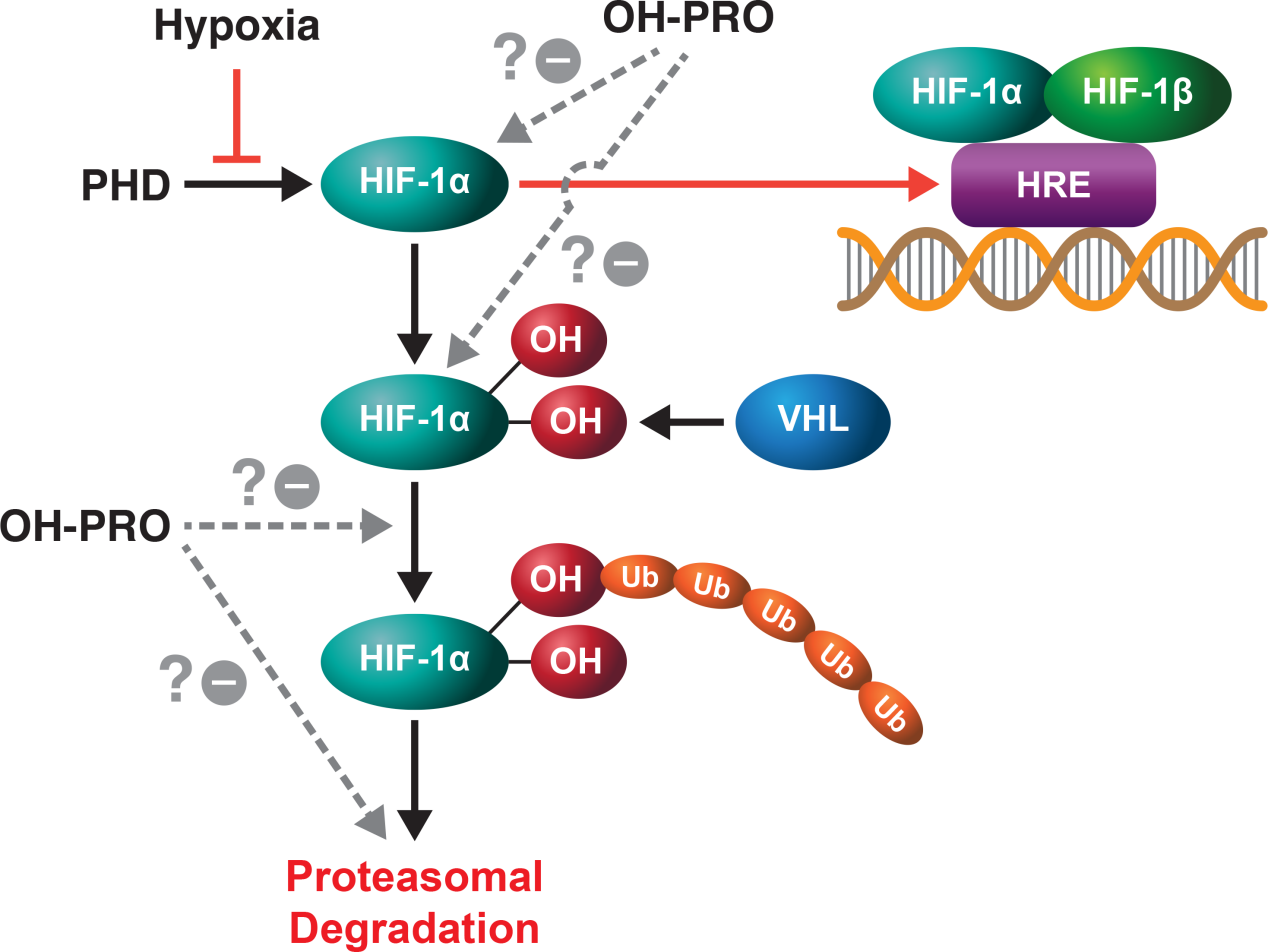
In conjunction with hypoxia, hydroxyproline increases the level of HIF-1α. Hydroxyproline augments the effects of hypoxia by increasing the levels of HIF-1α, but the site and mechanism for this effect has not been defined. It is possible that OH-PRO interacts at the HIF-1α hydroxylation step, at the recognition of hydroxylated HIF-1α by VHL or at the step of proteasomal degradation.

Recent work performed on hepatocellular carcinoma has shown that proline and hydroxyproline are linked to the HIF-1α pathway (**Figure 3**) (13). The limiting step in proline synthesis from glutamine, ALDH18A1 is upregulated with hypoxia and importantly, synthesized proline is incorporated into collagen, hydroxylated and released as free hydroxyproline. This was found to increase HIF-1α levels to increase tumor survival. Whether this effect on HIF-1α is due to hydroxyproline or OH-P5C will require additional studies. But the mutual cross-inhibition of OH-P5C and P5C make possible the proposal of a proline-collagen-hydroxyproline axis which interacts not only with metabolic epigenetic mechanisms but also with the HIF pathway, the main response to hypoxia.

## The relationship of glutamine and proline metabolism

The renaissance of metabolism in cancer was based on the seminal discoveries of Otto Warburg during the 1920s (1). He described the metabolic phenomenon in cancer as oxidative glycolysis. In the upsurge of metabolic oncogenesis during the 1990s, workers showed that an important reprogramming in metabolism was to spare carbons for the demands of increasing cell mass in carcinogenesis (30). Additionally, glutamine was thought to replenish the TCA cycle as well as supplying the nitrogen for nucleotide synthesis. Since the 1970s, it was recognized that glutamine was the medium of exchange for supplying carbons and nitrogen (31); excess protein was converted to glutamine in muscle and glutamine was used to supply growing tissue (intestine) as well as to function in acid-base balance (32). Little emphasis has been placed on the conversion of glutamine to proline, but convincing evidence from the relationship of proline to human cancer (33) together with the role of collagen synthesis makes it a critical agent for reprogramming. Additional work has to be done to reproduce the findings connecting proline metabolism to epigenetics through collagen synthesis and the dioxygenase mechanisms for demethylation and to the response to hypoxia through hydroxyproline turnover.

## Summary

Although the regulatory functions of proline and pyrroline-5-carboxylate was first described in 1985 (4), and the phenomenological relationships of proline degradation as a reservoir of substrates in collagen and the synthetic mechanisms are related to reprogramming at the DNA and histone level (8) as well as in the response to hypoxia (13), there was no convincing evidence for the mechanisms relating proline metabolism to the survival of cancer cells. Advances during the last several years have provided suggestive evidence that these interpretations are plausible. Some of the models are speculative at present but enough evidence exists to justify their introduction and projects to provide convincing evidence. We mow propose the following: l) PYCR3 is linked to the pentose phosphate pathway for the formation of PRPP and the maintenance of ribonucleotides as well as pyridine nucleotides; 2) synthesized proline is preferentially incorporated into collagen and its hydroxylation competes for the co-substrates of the dioxygenases necessary for demethylation of DNA and histones as a molecular mechanism for reprogramming; 3) the proline/hydroxyproline pathway augments the response to hypoxia; 4) the proline synthetic pathway plays a critical role in the pluripotency of CSC; 5) although contributions to the TCA cycle is an important function of glutamine, the proline/collagen/hydroxyproline regulatory axis may be the most important regulatory function for the metabolism of glutamine.

## Author contributions

The author confirms being the sole contributor of this work and has approved it for publication. Enquiries about this article can be sent to Dr. Lino Tessarollo (essarol@mail.nih.gov).
